# Rectification: the well-known “hand with gunshot” radiograph signed by M.I. Pupin (1858–1935) and published in 1931 is a forgery

**DOI:** 10.1055/a-2742-1763

**Published:** 2025-12-17

**Authors:** Jean-Francois Monville, Robert Ferdinand Dondelinger

**Affiliations:** 1 Medical ImagingSankt Nikolaus HospitalEupenBelgium

**Keywords:** M.I. Pupin, intensifying screen, fluorescent screen, Columbia College - New York, Prescott Hall Butler, X-ray hand with gunshot

## Abstract

**Background:**

The well-known “hand with gunshot” X-ray bearing the signature of M.I. Pupin from Columbia College New York is widely recognized in the literature as being the first radiograph acquired with the use of an intensifying screen.

**Method:**

Personal research in the New York City daily newspapers from 1896 and at Columbia University allowed us to establish that Pupin took two X-rays of a “hand with gunshot” in a patient on February 15 and 19, 1896. The first X-ray is preserved among the Pupin papers at Columbia University, and the second radiograph was published in an American magazine in March 1896. In 1974, the original radiographic plate of the X-ray performed on February 19 was discovered. In 1930 or shortly before, Pupin signed a contemporary X-ray of a “hand with gunshot”, which is incompatible with the historical X-rays. This X-ray was made public in 1931,1965, and 1969, with the false claim that it was the first X-ray acquired using an intensifying screen in 1896.

**Conclusion:**

The “hand with gunshot” X-ray signed by Pupin around 1930 is a crude forgery of the X-ray he made on February 19, 1896. This X-ray has since been published many times and has been erroneously taken in good faith for the original. The historical X-rays remained ignored.

**Key Point:**

- This paper establishes that the renowned “hand with gunshot” X-ray signed by M.I. Pupin is a forgery.

**Citation Format:**

- Monville J-F., Dondelinger RF. Rectification: the well-known “hand with gunshot” radiograph signed by M.I. Pupin (1858–1935) and published in 1931 is a forgery. Rofo 2025; DOI 10.1055/a-2742-1763

## Zusamenfassung

**Hintergrund:**

Die wohlbekannte Röntgenaufnahme „Hand mit Schrotkugeln“, mit der Unterschrift von M.I. Pupin vom Columbia College New York, ist in der Literatur weit verbreitet als das erste Röntgenbild, das mit Hilfe einer Verstärkerfolie erstellt wurde.

**Methode:**

Persönliche Nachforschungen in den New Yorker Tageszeitungen von 1896 und an der Columbia Universität haben erbracht, dass Pupin zwei Röntgenaufnahmen der Hand mit Schrotkugeln eines Patienten jeweils am 15. und 19. Februar 1896 erstellt hatte. Die erste Aufnahme ist Bestand Pupin’s Nachlasses der Columbia Universität; die zweite Aufnahme wurde in einer amerikanischen Zeitung im März 1896 abgebildet. Die Original-Platte der Aufnahme vom 19. Februar wurde 1974 entdeckt. Im Jahre 1930 oder kurz vorher unterzeichnete Pupin ein gegenwärtiges Röntgenbild einer „Hand mit Schrotkugeln“, die mit den historischen Aufnahmen von 1896 unvereinbar ist. Dieses Röntgenbild wurde 1931, 1965 und 1969 in Amerika bekannt gemacht mit der falschen Behauptung, es handle sich um die erste Röntgenaufnahme, die mit Hilfe einer Verstärkerfolie 1896 erstellt wurde.

**Schlussfolgerung:**

Die Röntgenaufnahme um 1930 mit der Handschrift von Pupin ist eine grobe Fälschung seiner historischen Aufnahme vom 19. Februar 1896. Dieses Röntgenbild wurde viele Male im guten Glauben anstelle des Originals gedruckt. Beide historischen Aufnahmen bleiben unbeachtet.

**Kernaussage:**

- Unsere Studie erbringt den Beweis, dass die wohlbekannte, von M.I. Pupin handsignierte und um 1930 erstellte Röntgenaufnahme „Hand mit Schrotkugeln“, eine Fälschung ist.

## The contemporary background

The news of the discovery of Röntgen rays was published in Vienna, Austria, by Die Presse on January 5, 1896 1 . The London Daily Chronicle reported the news on January 6 2 . A dispatch was published by The Sun (New York) on January 7 3 followed by The Journal (New York) the next day 4 . The Electrical Engineer (New York) shared the message on January 8 5 . Illustrated Electrical Review printed a copy of the article from The Sun on January 15 6 . Electricity issued a disbelieving editorial on January 22 7 . The Electrical World published an article on January 25 8 . The Electrical Worker 9 and The Photographic Times 10 printed a version of the story in March. In his preliminary communication, Wilhelm Conrad Röntgen (1845–1923) used 84 words to describe the production of X-rays 11 . The notice delivered by The Sun cut down Röntgen’s account to 44 words. It was not essential for American physicists to waiting for the translation of Röntgen’s publication to begin using X-ray photography. Röntgen’s original article, copied from a translation printed in Nature on January 23 12 , was circulated in the United States (US) by Electricity on February 5 13 , The Electrical Engineer on February 12 14 , and Science on February 14 15 . It is generally accepted that the first documented intentional X-ray in the US was acquired by Arthur Williams Wright (1836–1915) on January 27 at Yale University, New Haven, Connecticut 16 . Edwin Brant Frost (1866–1935) from Hanover, New Hampshire took the first X-ray to be applied to diagnosis in medicine in the US on February 3 17 . John Cox (1851–1923) and Doctor Robert Charles Kirkpatrick (1863–1897) from Montreal, Canada, were the first to use X-rays as a guide for surgery in North America on February 7 and 8 18 . In Chicago, Illinois, electrical engineer Charles Ezra Scribner (1858–1926) and Doctor James Burry (1853–1919) provided evidence of buckshot between the metacarpal bones in a patient on February 11 19 . The foreign body was extracted under the guidance of the roentgenograph on February 13 20 . The Journal reported on February 9, 1896 that a revolver bullet and a needle located in a hand were surgically removed based on X-rays in Europe 21 . The needle was extracted in Munich, Bavaria, before February 6 and the bullet was documented in Elberfeld, Rhineland, before February 3 22 . Other roentgenographic demonstrations of foreign bodies lodged in the hand or foot followed by surgical extraction were performed elsewhere, notably in England before February 13, 1896 23 24 .

## M.I. Pupin started experiments with Röntgen rays on February 1, 1896

**Fig. 1:**
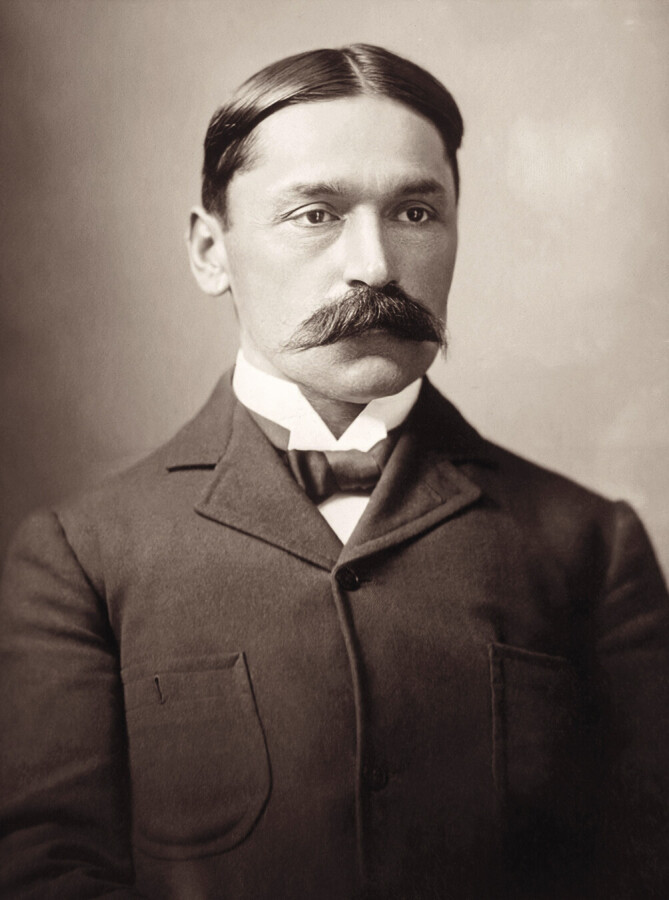
Portrait photograph of Michael Idvorsky Pupin (1854–1935) professor at Columbia College. (personal collection) [rerif]

In 1896, the physicist Mihajlo (Michael) Idvorsky Pupin (1858–1935) was an adjunct professor of electro-mechanics at Columbia College, New York ( Fig. 1 ). In his first article devoted to Röntgen rays, published on February 5, Pupin discussed theoretical aspects of Röntgen’s discovery 25 . Pupin started experiments with Röntgen rays on February 1. He designed electrodeless X-ray tubes, thus avoiding overheating. On February 4, he imaged a distinct shadow of a key and a corkscrew 26 . On February 5, selected objects and a human hand were radiographed 27 . In a paper published on February 12, Pupin wrote that he had succeeded in repeating Röntgen’s experiments a few days after February 5. A clear X-ray of various objects in an aluminum box was shown. The exposure time was one hour. While looking for a method of reducing the time of activation of the Crookes tube, Pupin suggested the use of a fluorescent screen: *“A fluorescent screen placed in front of the sensitive plate for the purpose of shortening the time of exposure gave encouraging results”* 28 . In a paper published on February 14, Pupin confirmed that he succeeded in repeating some of Röntgen’s experiments around February 8 29 . In an article published on April 15, Pupin cited February 7, as the date on which he had used a fluorescent screen for the first time 30 . Many years later, Pupin claimed that it was February 6, according to personal notes 31 . In the articles of February 12 and 14, Pupin suggested replacement of the Ruhmkorff (1803–1877) induction coil with a static Wimshurst (1832–1903) or Holtz (1836–1913) influence machine. The Galvano-Faradic Company of New York made a six-plate Holtz machine available to Pupin. The College of the City of New York provided Pupin with a pear-shaped Crookes tube with a small focusing spot. Pupin reported on these advances in a paper published on February 19 32 . Concerning the suggestion of using a fluorescent screen for X-ray photography, Pupin never published a demonstrative picture nor comparative X-rays on purpose. However, demonstrative comparative results were obtained with some unrevealed fluorescent substance by another contemporary English native pioneer, John Carbutt (1832–1905), who relocated to Philadelphia, Pennsylvania and was an expert in photographic processing and materials. His X-rays were exhibited in public on February 21, 1896 33 . The editor of Electricity explained that Pupin covered the photographic film with a sheet of paper that was dipped into a solution of sulfate of quinine. This innovation was the first specific mention of a fluorescent substance used as a kind of intensifying screen in the US. The novelty was declared working satisfactorily by the editor of Electricity [28 bis]. In early Spring 1896, a controversy was brewing in New York City regarding Pupin’s claim of being the first (in the US) to employ a fluorescent screen in Röntgen photography. The editor of Electricity backed up Pupin’s claim 34 . However, The Electrical World was not enthusiastic about Pupin’s results obtained in February: *“Dr. Pupin tested the effect of treating the film of the negative with a fluorescent substance, which treatment appeared to increase the effect, though not so markedly as to permit any definite conclusion to be drawn”* 35 . In Europe, researchers had a similar experience with the use of fluorescent substances for X-ray ahead of Pupin’s suggestion on February 12. Among these was Friedrich Eberhard Gieseler (1839–1921), professor at the agricultural academy of Bonn-Poppelsdorf, Germany, who found around January 24, that iron chloride best enhanced the photographic action of the X-rays on the plate 36 . In Pisa, Italy, the physicists Angelo Batelli (1863–1916) and Antonio Garbasso (1871–1933) had used fluorescence for the same purpose before January 25, 1896 37 . Alan Archibald Campbell Swinton (1863–1930), a London X-ray pioneer, informed Nature on April 22 that he suggested the use of a suitable fluorescent material as early as January 30 38 , and Charles Henry (1859–1926) in Paris used phosphorescent zinc chloride to increase the photographic effect of Röntgen rays before February 10, 1896 39 .

## Two forgotten X-rays of a “hand with gunshot” acquired on February 15 and 19, 1896

**Fig. 2:**
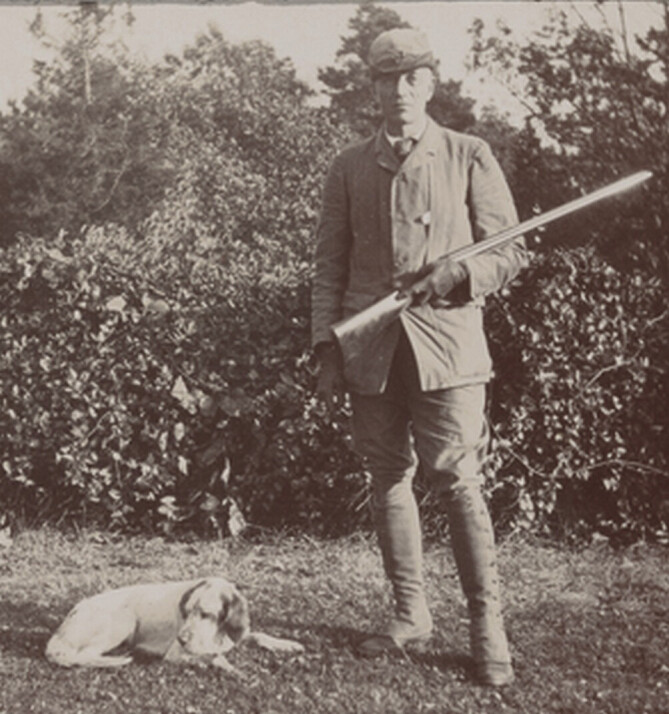
Photograph of Prescott Hall Butler (1848–1901). Manuscripts and Archives Division. The New York Public Library. “Prescott Hall Butler, St. James, New York”. Digital Collections. Accessed 05.11.2024. ( https://digitalcollections.nypl.org/items/54ec3d20-c52d-013c-561d-0242ac110003 ) [rerif]

**Fig. 3:**
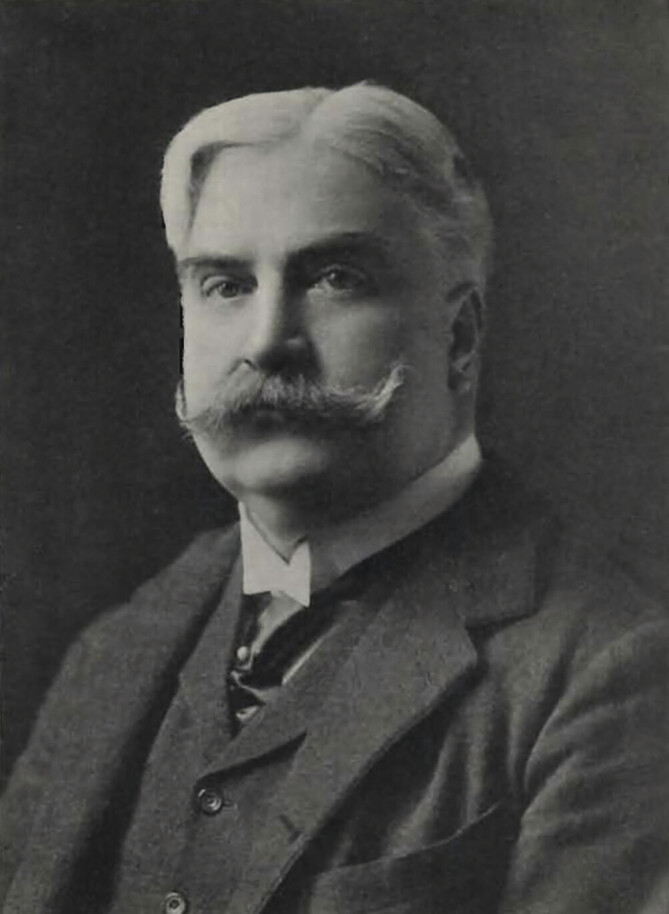
Portrait photograph of William Tillinghast Bull (1849–1909). Columbia University Archives, New York. Historical Photographic Collection, 1858-. Box 14.[rerif]

Prescott Hall Butler (1848–1901) ( Fig. 2 ) was a prominent New York lawyer 40 . At the end of 1895, Butler was the victim of a self-inflicted hunting accident. Discharge from a ground cannon was shot into his left hand. According to Pupin’s autobiography, the patient consulted Doctor Bull (1849–1909), an eminent New York surgeon 41 42 ( Fig. 3 ). Butler was sent to Pupin to obtain an X-ray of his hand prior to surgical extraction. On February 16, The New York Tribune wrote that Butler visited Pupin on February 15, but Pupin could not oblige, as he did not have a high-vacuum Crookes tube ready. Pupin postponed the experiment. However, the physicist did acquire X-rays on February 15 with an ordinary vacuum tube and an exposure time of one hour 43 . Over the course of February 16, Pupin obtained images of a human hand using the static machine and high-vacuum tubes. Exposure times of two hours were reported by the press. No use of a fluorescent screen was mentioned 44 . In the evening of February 17, Pupin attended a conference with hospital managers who envisioned erecting a radiographic facility 45 . On February 18, Pupin signed an extensive article on the radiographic technique, to be published in March 46 . The use of a fluorescent substance to shorten the exposure time was not referenced. Pupin did not mention Butler’s first visit to Columbia College. Butler returned to Pupin’s laboratory for the second time on February 19. A press article stated: *“Professor Pupin subjected the hand to an hour’s exposure …”* 47 . The use of a fluorescent screen was not mentioned and the exposure time was not shortened. The New York Tribune on the next day included an additional article on Butler’s X-ray which reported the following: *“On the previous days* [i.e. February 15] *, it will be remembered, he photographed a human hand, in which were imbedded no fewer than twenty-four shot … The bones were not as distinctly shown as Dr. Pupin wished. Consequently, yesterday* [February 19] *Prescott Hall Butler, whose hand it was … consented to the radiance for a second time. This second plate not only clearly outlined each bullet, but also with equal clearness defined the bones of the hand”* 48 . The column reminded readers that Butler had received an unsatisfactory X-ray on February 15, but with a common non-high-vacuum tube. The X-ray performed on February 19 was finally adequate. On the evening of February 21, Pupin gave a lecture on Röntgen rays to the members of the School of Mining of Columbia College 49 . According to the press, the usefulness of a fluorescent screen was not emphasized by the presenter. In his autobiography published in1923, Pupin recalled Butler’s X-ray as follows:

*“… I obtained the first X-ray photography in America on January 2,1896. … Doctor Bull … sent me a patient with nearly a hundred small shot in his left hand. His name was Prescott Hall Butler … He was in agony … Mutual friends begged me to make an X-ray photograph of his hand and then enable Doctor Bull to locate the numerous shot and extract them. The first attempts were unsuccessful, because the patient was too weak … to stand photographic exposure of nearly an hour … Thomas Edison had sent me several excellent fluorescent screens. I decided to try a combination of Edison’s fluorescent screen and the photographic plate. The fluorescent screen was placed on the photographic plate and the patient’s hand was placed upon the screen. The X-ray acted upon the screen first and the screen by its fluorescent light acted upon the plate … A beautiful photograph was obtained with an exposure of a few seconds … Doctor Bull operated and extracted every one of them in the course of a short and easy surgical operation … That was the first X-ray picture obtained by that process during the first part of February 1896, and it was also the first surgical operation performed in America under the guidance of an X-ray picture … Nobody gives me any credit for the discovery, although I described it in the journal Electricity of February 12,1896 before anybody had even thought of it …”*^50^ .

**Fig. 4:**
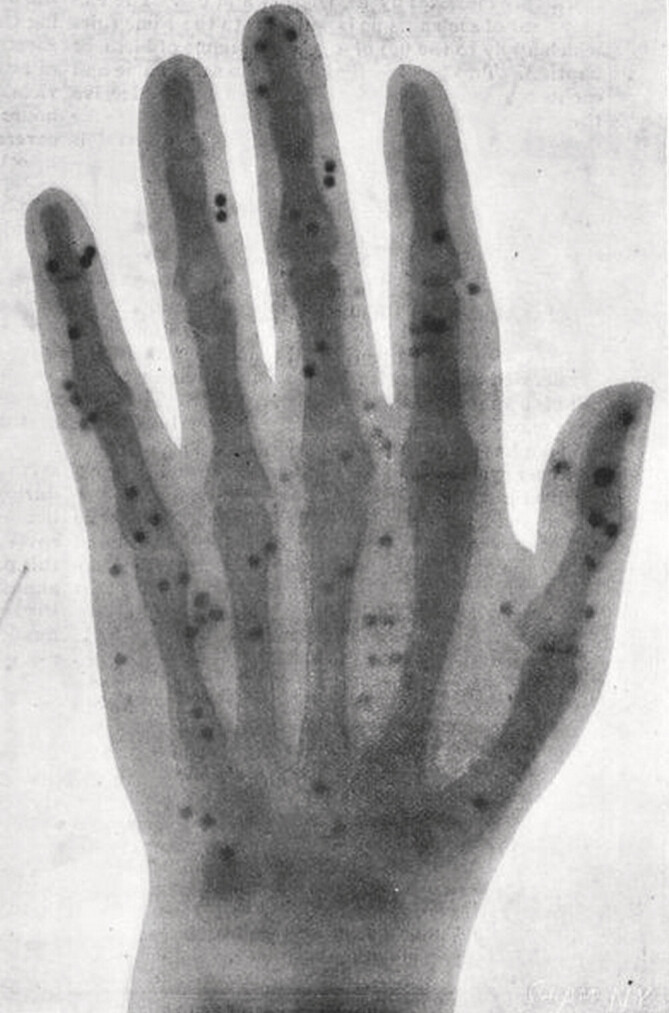
Print of the X-ray of Prescott Hall Butler’s left hand containing about 72 pellets, taken by M.I.Pupin at Columbia College, New York, on February 19,1896. Dr. Pupin’s X-ray photograph of a wounded hand. Scientific American, 21 March, 1896: LXXIV(12):184) [rerif]

On April 14,1896, Pupin contracted dreadful pneumonia, as did his wife, Sarah Catharine Jackson (1859–1896) who died on April 25. Pupin was left with distressing memories from the horrendous spring of 1896: *”… I must confess that I never returned to X-ray research, because for a long time after my illness, even the sight of an X-ray tube made me almost hysterical.* ” Pupin’s emotionally strained memory was erroneous when recalling facts from the year 1896. The incorrectness of Pupin’s memory is demonstrated when he claimed having produced his first X-ray on the unrealistic date of *“January 2, 1896”* . This late claim was widely accepted in the American literature, even though Pupin mentioned the date of February 1, 1896 in communications from 1896 51 . The description of the clinical status of Hall Butler is hyperbole. The patient was not *”in agony”* , as he came twice on foot to Pupin’s laboratory. The agony might have been a local complication from the pellets in the hand. Pupin obtained Butler’s X-rays on February 15 and 19, not *“during the first part of February* ”. The reported shortening of exposure time to *“a few seconds”* was another exaggeration, as was the number of *“nearly a hundred small shot.”* Contemporary newspapers reported a duration of exposure lasting one hour 47 or 20 minutes 52 . 72 or 77 shot were counted. In his autobiography, Pupin alluded to a dramatic scenario, in which the idea of applying the fluorescent screen to the photographic plate had come to his mind all of a sudden: *“I decided to try a combination of Edison’s fluorescent screen and the photographic plate”.* Pupin neglected to reveal that he had tried sulfate of quinine and platino-barium cyanide as X-ray intensifiers before. Pupin neglected to say that he had already acquired an X-ray of Butler on February 15, but it was unreadable. The surgical treatment occurred after February 19, if it ever took place. It is reasonable to assume that Dr. Bull did not extract *“every one of* [the pellets] *in the course of a short and easy surgical operation”* . Presumably the surgeon left in place most of about twenty-eight silent shot imbedded in the palm, according to standard surgical practice. By all evidence given in the first chapter, the extraction of pellets from Butler’s hand was not *“the first surgical operation performed in America under the guidance of an X-ray picture”.* The X-ray of Butler’s “hand with gunshot” from February 19, was published on March 21 52 ( Fig. 4 ). The film is of average quality for the time. No fluorescent screen for shortening exposure time was mentioned. 72 shot were counted. Pupin used a classical spherical Crookes tube and a powerful induction coil. This unique paper print went unnoticed.

## An X-ray of a “hand with gunshot” signed by Pupin was published in 1931

**Fig. 5:**
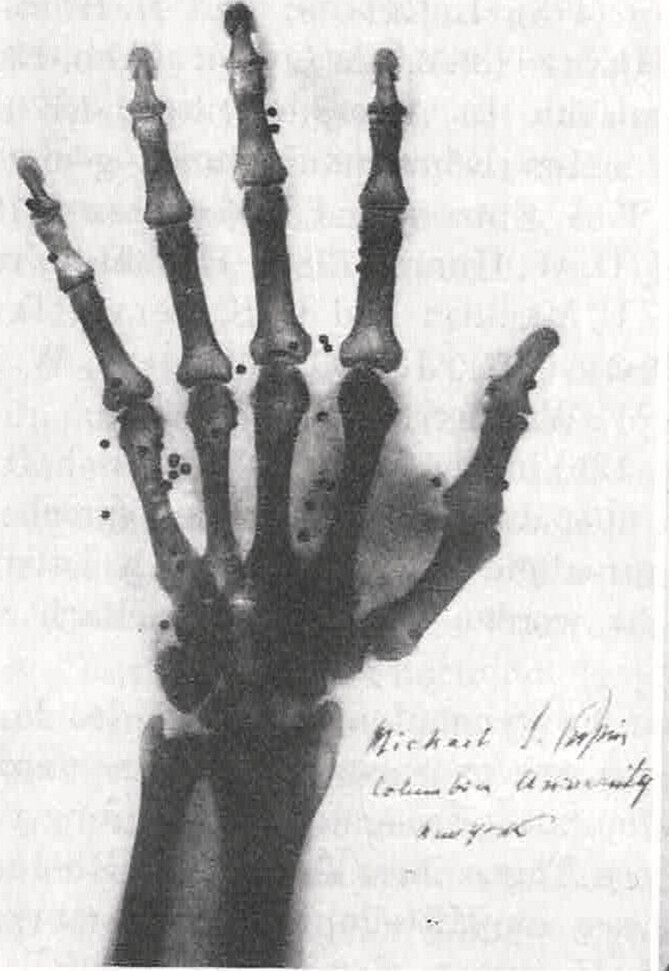
A forgery of the “hand with gunshot” X-ray published in Röntgen’s biography by Glasser in 1931. Reproduced as published. Glasser O. Wilhelm Conrad Röntgen und die Geschichte der Röntgenstrahlen. Berlin, Verlag von Julius Springer, 1931:27 [rerif]

In his authoritative biography of Röntgen and the description of the early history of radiology, Otto Glasser (1895–1964) published in 1931 an X-ray of a “hand with gunshot”, bearing Pupin’s undated signature [31 bis] ( Fig. 5 ). The caption read: “ *Hand with gunshot. Produced by M. Pupin in New York during February 1896”.* Glasser made no mention of the use of an intensifying screen by Pupin, but he commented elsewhere that Pupin was recognized in the US as the inventor of the intensifying screen [31 ter]. The “hand with gunshot” radiograph shown together with other roentgenographs obtained by American and European pioneers during January or February 1896, went without comment, despite reproduction in the English translation and all subsequent editions of Glasser’s work. Obviously, Pupin himself had made the radiograph available for publication. Indeed, in a letter dated 25 February 1930 [31 quarter], Pupin stated that he retained no more early X-rays of his own, except the film of the hand of Prescott Hall Butler: *“… the X-ray of the late Mister Prescott Hall Butler is still in my possession. This X-ray was obtained with the assistance of the intensifying screen and is an excellent picture …”* Pupin further reminded Glasser of his description of the fluorescent screen in Electricity on February 12, 1896. However, even by a superficial comparison with the X-ray published on March 21, 1896, it is obvious that the X-ray that appeared some thirty-five years later is a gross forgery of the original. Why did Pupin send an autographed falsification of a personal X-ray to Glasser? Most likely, because the physicist was no longer able to access the original plate or print in 1930, despite his affirmation, but he wanted the X-ray to be published as a testimony to his invention of the intensifying screen in February 1896.

## The same “hand with gunshot” X-ray surfaced again in 1965 and 1969

The Pupin-Butler X-ray anecdote of 1896 vanished rapidly into obscurity. Neither the episode described by Pupin in his autobiography in 1923 nor the publication of a fake of Pupin’s original X-ray in 1931 received much attention.

**Fig. 6:**
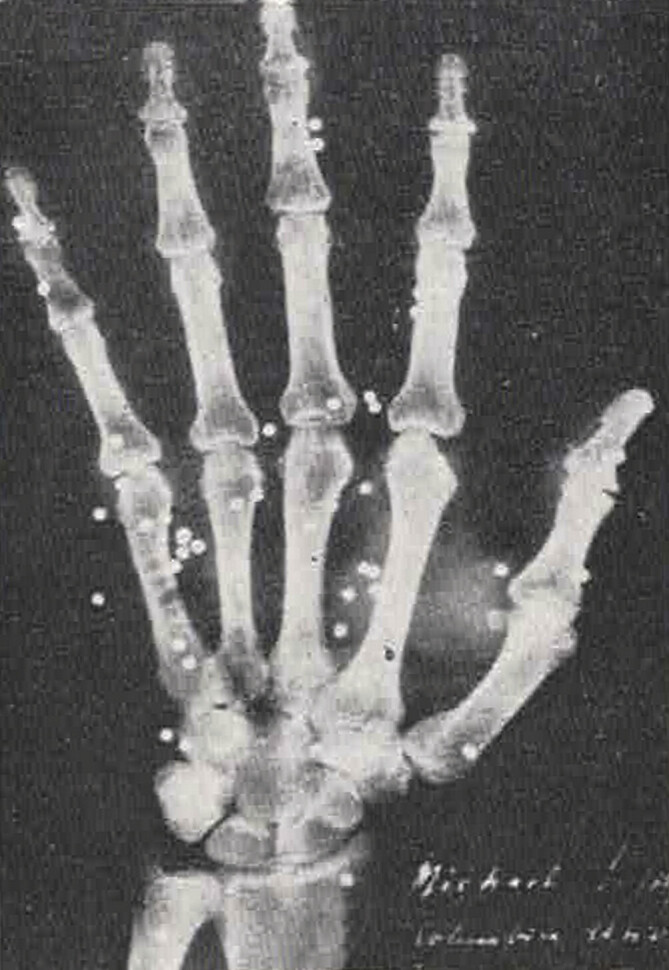
The same forgery as shown in Figure 5, published by Grigg in 1965. Reproduced as published. Grigg, Emanuel R.N.: The Trail of the Invisible Light. Springfield, Illinois, Charles C. Thomas,1965:30 [rerif]

In his abundantly documented book on the history of radiology published in 1965, Emanuel R.N. Grigg (1916–1976) reproduced the same fake image as a positive print ( Fig. 6 ) [51 bis]. The author commented erroneously: *“… This is the famous gunshot-ridden hand of Prescott Hall Butler, which Pupin examined on February 14, 1896. The print is so much better than anything available at that time, one would be inclined to regard it as a forgery – but it is authentic and had been reproduced in the Scientific American of March 21,1896”.* The date of February 14 is incorrect. Obviously, the author was struck by the outstanding quality of an X-ray taken in February 1896, but he did not identify that it was counterfeit, thereby unintentionally misleading the readers. Grigg further informed readers that on August 5, 1933, Pupin sent a letter to Dr. Maximilian Hubeny (1880–1942), the former editor of Radiology and in 1933 Chairman of the History Committee of the first American Congress of Radiology, which was to meet in Chicago, Illinois in autumn and included the four US radiological societies 53 . Pupin attached a print of the falsification published in 1931 and commented:

*“My dear Doctor Hubeny: I send you the X-ray picture taken by myself in February 1896. It was the first picture to guide a surgical operation in the United States. I also enclose a brief account of this picture and the discovery of the method of shortening the time of exposure in X-ray photography. The description was copied from 307–308 of my autobiography ‘From immigrant to inventor’. Yours Sincerely, (ss) M.I. Pupin.”*

Pupin was still upset in 1933 that he had received no credit for suggesting the use of the intensifying screen in 1896 despite publication of a forgery in 1931. He again sought recognition for the first use of a fluorescent screen in 1896, but none of the American radiological societies pursued the matter.

**Fig. 7:**
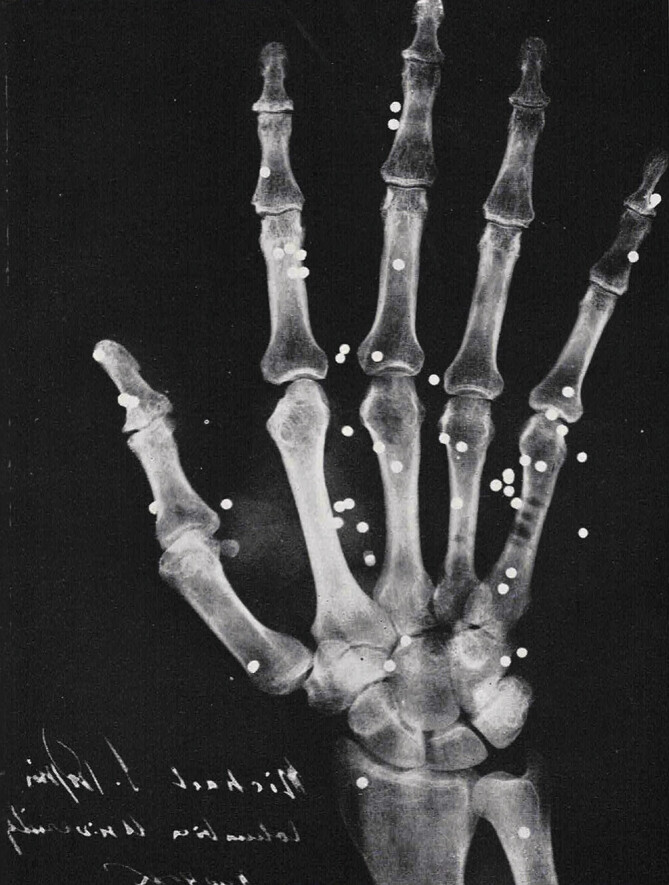
X-ray of the left hand of an anonymous individual, signed by Michael I. Pupin. Reproduced as published. Brecher R. and E. The Rays. A History of radiology in the United States and Canada. Baltimore,The Williams and Wilkins Company,1969:Plate VI. [rerif]

In 1969, Ruth (1911–1966) and Edward (1911–1989) Brecher published their history of radiology in the United States and Canada after being commissioned by the American College of Radiology in 1963. Pupin’s forgery was shown on the first pages as a negative, the sides were reversed ( Fig. 7 ). The caption erroneously said: *“Plate VI. From American Institute of Radiology. X-ray of hand with buckshot made by Professor Michael Pupin in February 1896. See page 56”* 54 . The authors were aware that the X-ray was not acquired during the first part of February 1896 and was not the first X-ray serving as a guide to a surgical operation in America [54 bis]. However, they did not discover the falsification.

## Discoveries related to Butler’s “hand with gunshot” X-ray up to the present

**Fig. 8:**
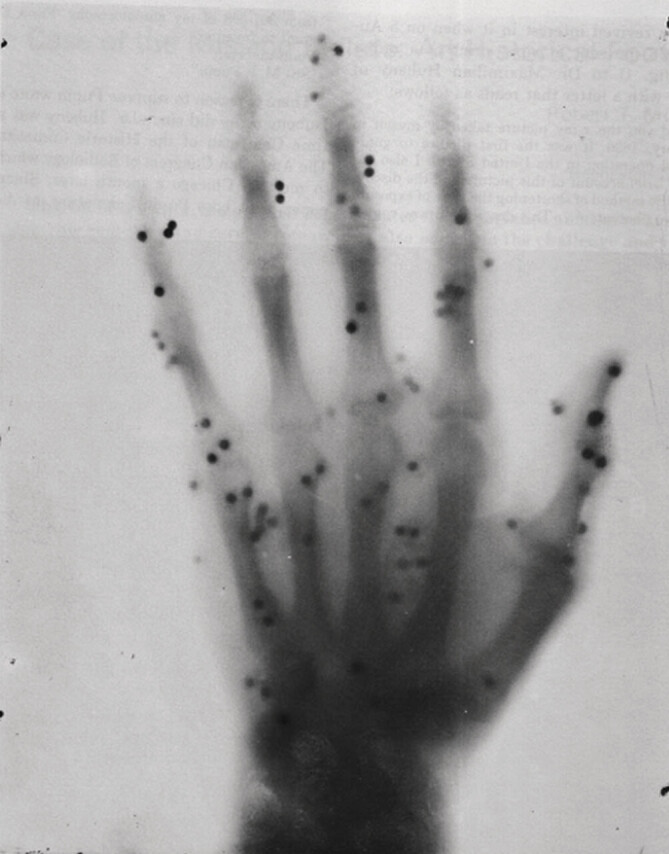
Original photographic plate of the X-ray of Prescott Hall Butler’s left hand containing 77 recognized pellets taken by M.I.Pupin at Columbia College, New York, on February 19,1896. Morrison, M.T. The case of the missing pellets: a historical footnote. Radiology Technology,1974;45(5):328–332 [rerif]

In 1974, Robert Thomas Morrison (1897–1980), who was appointed curator of the American College of Radiology Foundation in 1964 55 , discovered the original photographic plate of Butler’s film, which was in undisclosed private hands 56 ( Fig. 8 ). The plate is superimposable in all respects on the print published in Scientific American. 77 pellets clearly appear on the plate. Both the plate and the published print are definitely from the X-ray taken by Pupin of Butler on February 19,1896.

**Fig. 9:**
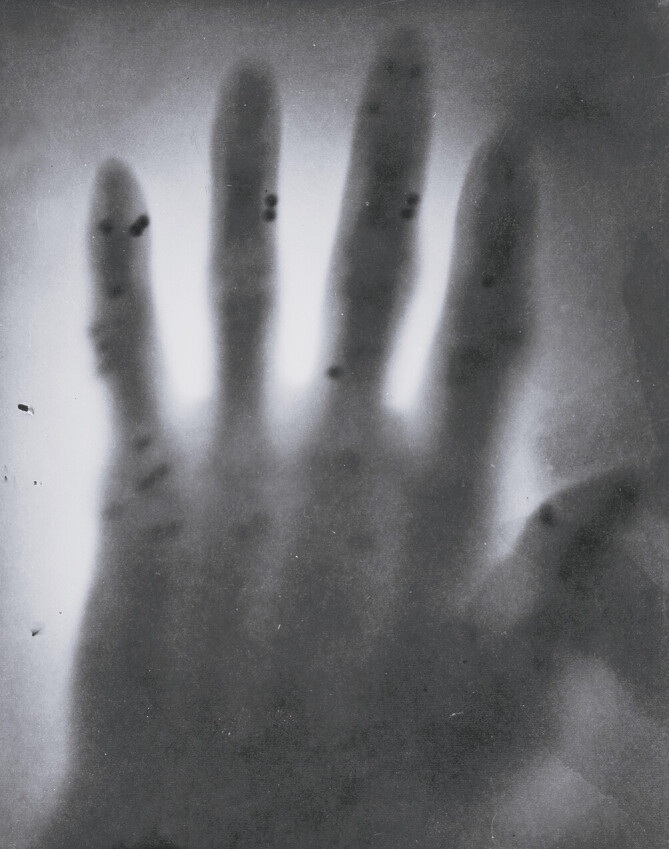
Print of the X-ray of Prescott Hall Butler’s left hand with gunshot acquired by Pupin at Columbia College, New York, on February 15,1896. Michael Idvorsky Pupin Papers. Rare Book & Manuscript Library, Columbia University in the city of New York. Available from: https://exhibitions.library.columbia.edu.exhibits/show/jewels/themes/science170 [rerif]

The hazards of history led to Columbia University becoming the guardian of an X-ray of a “hand with gunshot”, which is the only X-ray stored among the Pupin papers 57 ( Fig. 9 ). It is a printed copy of a radiographic plate mounted on cardboard. A pencil annotation reads: *“sometime after January 1896”* (personal communication dated 04/20/2020 by Mrs. Jennifer Lee, former curator of the Performing Arts Collection, Butler Library, Columbia University). The radiographic quality is poor due to underexposure. However, it is unquestionable that the individual of whom this X-ray was taken is Prescott Hall Butler. All detectable pellets are located exactly in the same position as on the X-ray in Fig. 4 and Fig. 8 . The hand was not positioned flat on the photographic plate. Pupin reported that he took two films of Butler, but he let the reader believe that both X-rays were taken the same day. Unfortunately, on February 15, a film of acceptable photographic quality was not achieved. The most probable reason for the failure was an insufficiently exhausted X-ray tube. The poor-quality film, which is preserved at Columbia, is the result of the exposure on February 15. The X-ray published in Scientific American is the result of the exposure on February 19.

The forgery has been erroneously accepted until now in good faith by all as authentic. From the comparison of the X-rays shown in Fig. 5 , Fig. 6 , and Fig. 7 to Fig. 4 and Fig. 8 , it is clear that the bone anatomy of the radiographed hand, the position of the digits, the number of buckshot, the standard of radiographic technique, and the artifacts differ. One can also notice that the distal forearm with two pellets is included in Fig. 5 , Fig. 6 , and Fig. 7 but is not shown in Fig. 4 and Fig. 8 . The X-ray published in 1931 is the only reproduction that shows a larger part of the bones of the distal forearm. Radiolucencies are clearly seen in the fourth and fifth metacarpal in Fig. 5 , Fig. 6 , and Fig. 7 , but they are absent in Fig. 4 and Fig. 8 . Twenty-three shot are missing on Fig. 5 , Fig. 6 , and Fig. 7 . Obviously, the X-ray shown in these figures is from a much later date than the film depicted in Fig. 4 and Fig. 8 . The comparable pellets shown on the two groups of X-rays are not exactly in the same location in relation to each other or the skeleton. Resemblance between the fake and the original with respect to the distribution of the pellets strongly suggests that the falsifier was aware of the picture published in Scientific American. However, the imposter was not careful, for whatever reason, resulting in the missing pellets. We made an inquiry to the Radiological Society of North America (RSNA) (personal communication dated 08/30/2024 by Mr. Mark Watson, Executive Director) and the American College of Radiology (ACR) (personal communication dated 09/22/2024 by Mrs. Dana Smetherhand, Chief Executive Officer) in an attempt to determine the repository of the forged X-ray print, but the brief search was unsuccessful. Grigg wrote in 1965 that the X-ray was stored at the department of radiology at Cook County Hospital in Chicago, Illinois which closed its door in 2002. Ruth and Edward Brecher mentioned in 1969 that the X-ray was preserved at the American Institute of Radiology. Ignoring the gossip 58 and sticking to the facts, we are cognizant of the fact that we will probably never know with certainty who commissioned the fraud, who committed it, and who knew about it. However, we do know that Pupin used the forgery to his advantage either with or without knowledge of the fraud.

## Conclusion

Professor M.I. Pupin from Columbia College, New York was the earliest X-ray pioneer in New York City and performed research from February 1 to April 14, 1896. On February 12, Pupin suggested the use of a fluorescent screen to shorten the exposure time. Pupin applied fluorescent chemical substances in different ways without achieving convincing results. On February 15, Pupin acquired a poor-quality X-ray of Prescott Hall Butler’s “hand with gunshot”. On February 19, he obtained a second X-ray of acceptable quality that was published in Scientific American on March 21. In his autobiography published in 1923, Pupin mentioned specifically the use of a fluorescent screen of platinocyanide of barium, made available to him by Edison. Neither contemporary press articles nor Pupin’s publications made an explicit reference to a fluorescent screen being employed during exposure of any X-ray published by Pupin, highlighting the fact that no particular benefit was achieved. A forgery of Butler’s X-ray, sent by Pupin to Glasser, was published in 1931 and was reproduced thereafter in two major books on the history of radiology, edited in the US in 1965 and 1969. The imitation has since been reproduced many times erroneously in good faith as an example of the outstanding radiographic quality achieved in 1896 by applying a fluorescent screen. In 1933, two years before his death, Pupin unsuccessfully attempted to be recognized for the discovery of the intensifying screen in the US, based on the counterfeit X-ray. Furthermore, he erroneously claimed to be the first to have produced an X-ray to serve as a guide for surgery in America. The origin and the circumstances of the fake remain unclear. Pupin can be given credit for being the first in the US to suggest the use of a fluorescent intensifying screen. However, his claim of being the first to apply an X-ray to surgery in the US or North America is unfounded.
